# Antibiotic removal does not affect cecal microbiota balance and productive parameters in LP robust rabbit line

**DOI:** 10.3389/fvets.2022.1038218

**Published:** 2022-11-07

**Authors:** Laura Montoro-Dasi, Laura Lorenzo-Rebenaque, Adrian Ramon-Moragues, Maria Teresa Pérez-Gracia, María de Toro, Clara Marin, Arantxa Villagra

**Affiliations:** ^1^Departamento de Producción y Sanidad Animal, Salud Pública Veterinaria y Ciencia y Tecnología de los Alimentos, Instituto de Ciencias Biomédicas, Facultad de Veterinaria, Universidad Cardenal Herrera-CEU, CEU Universities, Valencia, Spain; ^2^Centro de Investigación y Tecnología Animal, Instituto Valenciano de Investigaciones Agrarias, Castellón, Spain; ^3^Área de Microbiología, Departamento de Farmacia, Instituto de Ciencias Biomédicas, Facultad de Ciencias de la Salud, Universidad Cardenal Herrera-CEU, CEU Universities, Valencia, Spain; ^4^Plataforma de Genómica y Bioinformática, Centro de Investigación Biomédica de La Rioja, La Rioja, Spain

**Keywords:** rabbit production, antibiotic supplementation, farm management, microbiota, 16S rRNA analysis, livestock

## Abstract

Antimicrobial resistance is an important threat to public health worldwide, being one of the main death causes in 2050. Moreover, global health is currently underpinned by the “One Health” concept, whereby livestock is strictly related to human and environmental health. However, in the case of the meat rabbit industry, antibiotic additives are still added to prevent gastrointestinal diseases. Current food and consumer awareness require the implementation of sustainable production systems, where robustness and resilience are increasingly important. Hence, the aim of this study was to evaluate the effect of antibiotic feed supplementation on microbiota, and productive performance during the rabbit growing period in a robust genetic line. For this purpose, a total of 432 weaned rabbits were randomly housed, cecum samples were taken on the weaning day and at the end of the growing period (28 and 61 days of age, respectively), and 16S rRNA sequencing analysis was performed. Results showed a higher microbiota complexity at the end of growing in both experimental groups. Firmicutes represented the dominant phylum of the cecal community, followed by Bacteroidota in both groups. Moreover, *Victivallis* and *Escherichia-Shigella* genera were only identified in the experimental group without antibiotic supplementation at the end of the growing period. In conclusion, antibiotic feed supplementation had no effect on microbiota composition and productive performance in the robust genetic line reared. These results evidence the importance of the development of rabbit robust genetic lines as an alternative tool to antibiotic administration in epizootic enteropathy control.

## Introduction

Antimicrobial resistance (AMR) is an important threat to public health worldwide (1). In fact, 700,000 people die annually as a result of resistant bacteria around the world. The World Health Organization published that by 2050, AMR and consequent failed treatments will cause 10 million deaths and economic losses of $100 trillion annually (2). Moreover, global health is currently underpinned by the “One Health” concept, whereby livestock and agri-food systems are at the crossroads of human, animal and environmental health (3). In fact, a close association has been demonstrated between antibiotic (AB) use in animal production and the emergence of AMR in humans and environment, making it mandatory to reduce AB administration in farms (4, 5). However, in the case of the meat rabbit industry, although the use of AB as growth promoters has been banned in the European Union (EU) since 2006 (6), AB additives are still added to rabbit diets to prevent gastrointestinal disease (rabbit epizootic enteropathy) (7). In this sense, although humankind depends on agriculture and livestock for its food, more than 20% of current losses in animal production are still linked to animal diseases (8).

Moreover, as the world population is set to increase to 10 billion people by 2050, the animal protein required will be more than 70% of current yields (9). In this context, the rabbit has many interesting aspects for which it could be considered an ideal meat-producing animal: it has a short life cycle, a short gestation period and a high feed conversion ratio (10). This production sector is important in the EU, which is the world's second largest meat rabbit producer, accounting for 93% of the world's imports and exports, with Spain as one of the major exporting countries (11).

Metagenomic studies are therefore becoming increasingly relevant, due to the important role of microbiota balance in animal health, welfare and meat production (6). The gut microbiota is the complex microbial communities (bacteria, fungi, archaea, protozoa, and virus) that live together in a harmonic and dynamic equilibrium interacting with the host, playing an important role in metabolic, and immunologic functions (12). However, when this equilibrium is disturbed and beneficial bacteria cannot control detrimental bacteria, leads to in a dysbiosis status (13). Dysbiosis status has been related to pathologies and reduced production parameters. For that reason, many therapeutic strategies aimed at restoring the equilibrium of the intestinal microbiota (13). It is demonstrated that the presence of AB could affect the caecal microbial environment by modulating the microbiota composition and enhancing metabolic capacities by improving digestion and absorption of nutrients (7, 14, 15). Traditionally, antibiotics such as neomycin, tiamulin, valnemulin, chlortetracycline or bacitracin, have been used in rabbit production in sub-lethal doses to control pathogens and dysbiosis processes throughout the growing period (14, 16). Whitin them, neomycin and valnemulin are commonly used to prevent ERE, because it is demonstrated that increase rabbit's immune response, and modulate intestinal microbiota composition being specifically active against *Clostridium perfringes*, a pathogen strictly related to rabbit gastrointestinal disorders (17, 18). Nowadays, in rabbit production these molecules are still widely administered, especially after weaning, to control mortality peaks as a result of the onset of gastrointestinal symptoms (19). However, to be able to reduce AMR transmission throughout the food chain, new strategies should be developed to favor the establishing of a correct microbiota balance that increases the digestible efficacy of nutrients and maintains animal health and welfare (15).

Traditionally, strategies to meet protein demands were based on genetic selection focused on improving growth rate and muscle mass, and the intensification and automation of farm facilities (11, 20, 21). However, current food and consumer awareness require the implementation of sustainable production systems, respectful with animal welfare and efficiently facing environmental concerns (11). This objective is closely linked with genetic selection, where more rustic genetic lineages, which bear greater resilience against diseases, and therefore less AB requirement, will meet such concerns. Robustness is defined as the capacity to maintain adequate production levels, supporting all body functions at the highest performance, under different environmental conditions and production systems (22, 23). In accordance, the EFSA Panel on Animal Health and Welfare concluded that more emphasis should be placed on genetic selection traits such as disease resistance and stress resistance (11).

Nevertheless, to be able to assess the effectiveness of these alternatives it is necessary to have better knowledge of the development of microbiota composition with and without AB administration in broiler rabbits under animal production conditions (7). For this purpose, the cecum is commonly chosen to evaluate microbial composition and development, as it is the main site of fermentation and hosts the most diverse bacterial species of the gastrointestinal tract (7, 15, 19, 24).

Hence, the aim of this study was to evaluate the effect of AB feed supplementation on microbiota development (using 16S rRNA sequencing analysis) and productive performance during the rabbit growing period in a robust genetic line.

## Materials and methods

### Ethics statement

In this experiment, all animals were handled according to the principles of animal care published by Spanish Royal Decree 53/2013 (25). All protocols were approved by the Ethical Review Panel of the Directorate-General for Agriculture, Fisheries and Livestock of the Valencian Community under code 2018/VSC/PEA/0067.

### Experiment design

The study was carried out in the experimental farm of the Polytechnic University of Valencia (UPV, in its Spanish acronym, Valencia, Spain). All the animals were provided from the same center (UPV), from a robust line (called LP) created and developed there (26), selected for its resilience and its ability to make use of available resources (22, 27).

A total of 432 weaned rabbits (28 days of age) were randomly housed in 12 collective cages (36 animals/cage) of 100 x 75 x 50 cm in size until the end of the growing period (61 days of age). Moreover, two management conditions were evaluated: animals fed with AB supplementation (ABs group, 216 rabbits located in 6 collective cages), and animals fed without AB supplementation (NoABs group, 216 rabbits located in 6 collective cages). The house was supplied with programmable electrical lights 12L:12D, automated electric heating and forced ventilation, in line with common practice in rabbit production. The experimental pelleted diets were commercial feed according to standard diets for rabbits, and the only difference between them was the presence of AB (Table 1). Nutritional and product analysis was assessed before the arrival of animals. Feed was weighed, manually distributed and added *ad libitum*. Finally, the mortality and the presence of diarrhea were recorded daily.

**Table 1:** Analytical feed dry matter composition (%).

**Analytical constituents**	**Diet**	
	**ABs (%)**	**NoABs (%)**
Crude protein	14.50	14.50
Crude fat	2.90	2.90
Crude fiber	19.80	19.80
Crude ash	9.00	9.00
Calcium	1.30	1.30
Phosphorus	0.50	0.50
Sodium	0.25	0.25
Neomycin sulfate	250 ppm	-
Valnemulin hydrochloride	35 ppm	-

ABs, rabbits fed with antibiotic supplementation; NoABs, rabbits fed without antibiotic supplementation.

### Sample collection

In this experiment, two different sampling times were established: the weaning day (28 days of age) and the end of the growing period (61 days of age). At each sampling time, 4 animals per cage of each experimental group (*n* = 24 samples/ experimental group/ sampling time) were randomly selected and cecal samples were collected. Ceca were taken individually, placed in sterile jars and processed within 24 h after collection.

### Microbiota analysis

#### DNA extraction

In first place, cecal content was removed and homogenized. Then, pools of four animals from the same cage were prepared (*n* = 6 pools/ experimental group/ sampling time); the DNA of pools content was extracted (QIAamp Power Fecal DNA kit, Werfen, Barcelona, Spain) and frozen at −80°C for shipment to the Center for Biomedical Research of La Rioja (CIBIR, in its Spanish acronym, Logroño, Spain), according to the manufacturer's instructions.

#### 16S rRNA gene amplification and MiSeq sequencing platform

Once there, 16S rRNA gene amplification and MiSeq sequencing was performed according to Montoro-Dasi et al. (28) in the Centre for Biomedical Research of La Rioja (CIBIR, in its Spanish acronym, Logroño, Spain).

Briefly, primer sequences cover the V3–V4 regions of the 16S rRNA gene, and the following primers included the Illumina adapters: 16S Amplicon PCR Forward Primer = 50 (TCGTCGGCAGCGTCAGATGTGTATAAGAGACAGCCTACGGGNGGCWGCAG) and 16S Amplicon PCRReverse Primer = 50 (GTCTCGTGGGCTCGGAGATGTGTATAAGAGACAGGACTACHVGGGTATCTAATCC). Finally, the sequencing run was performed in a MiSeq (Illumina) system in 2 x 300 bp format. After evaluating the quality of the raw unprocessed reads, the adapters were removed, and the reads were re-evaluated. Finally, the V3–V4 region of the 16S rRNA gene was partially reconstructed into fragments of approximately 550–580 bp. The OTU (Operational Taxonomic Unit) picking, and analysis was performed with QIIME (v1.9.1) pipeline.

### Productive performance evaluation

To record performance data, animals and feed consumption were weighed per cage at weekly intervals. Thus, mean daily feed intake (ADFI), mean daily gain (ADG) and feed conversion ratio (FCR) were evaluated each week during the growing period.

### Statistical analysis

First, to evaluate performance parameters for all the observed variables, a descriptive analysis of each sample was carried out to detect out-of-range data (outliers), proceeding to eliminate those records that the program indicated as such. Then, a Kolmogorov-Smirnov test was performed to ensure data followed normal distribution. Finally, a multi-factor two ways Analysis of Variance (ANOVA) test was used to compare the performance results obtained for both experimental groups. Statistical Analysis was performed using the Statgraphics XVII Centurión^®^ program.

Furthermore, to perform the statistical analysis of bioinformatics results, demultiplexed paired FASTQ sequences were imported into the QIIME2 v2021.4. The DADA2 pipeline incorporated into QIIME2 was used for the denoising, filtering and chimera removal of the sequences and assigned reads into Amplicon Sequence Variants (ASVs). Then, taxonomic annotation was obtained using the SILVA v138 database (29) and sequences not assigned to any taxa or classified as *Eukaryote* or *Archaea* were filtered out. Sequencing statistical analyses were done using QIIME2 v2021.4. Moreover, to compare diversity and richness between cecal communities, Chao1, Shannon, and observed number of OTUs indexes were computed after OTUs rarefication at 17 658 contigs. The statistical method used for comparison of the communities was a paired samples analysis of variance that included the following factors: sampling time (weaning day *vs*. end of the growing period), AB treatment (ABs vs. NoABs). The significance of differences among different groups was evaluated by Kruskal–Wallis test. Box-and-whisker plots for species richness and evenness were generated using Graphad Prims 8. Finally, a Venn diagram was drawn up to show the shared and unique features among groups, based on the occurrence of features in a sample group regardless of their relative abundance, by using InteractiVenn software for Venn diagram construction (30). Finally, to quantitatively measure beta diversities, the Bray-Curtis distance and unweighted Unifrac and weighted Unifrac values were calculated, and Principle Coordinate Analysis (PCoA) plots were generated for the origin of the sample from Bray-Curtis distances using ClustVist software (31). Differences in microbial mean taxa abundance according to group were detected using ANCOM (Analysis of Composition of Microbiomes), with W value corresponding to the number of times an ASV abundance is significantly different for a group (32).

## Results

A total of 24 cecal pools were collected and processed: six cecal pools for each experimental group (ABs and NoABs) at the weaning day, and six cecal pools for each experimental group (ABs and NoABs) at the end of the growing period.

### 16S rRNA sequencing

The total of sequencing reads of the 24 cecal pools samples was 5 409 112 (mean 225 379.7 reads/sample), with a mean read length of 444.3 ± 13.88 pb. A total of 524,841 sequences and 5 091 ASVs were generated. A total of 6,001 chimeric sequences were removed from the dataset with a total of 518,840 sequences left for ASVs table generation and database alignment. After filtering, a total of 4,956 unique sequences were left for taxonomic assignment. The datasets generated and analyzed are available at NCBI's BioProject PRJNA612272 and BioSample SAMN30645692.

#### Bacterial diversity

The diversity study was performed after rarefying 17,658 reads, with the samples from the NoABs group at weaning day as the limit of the rarefaction. Diversity analysis for the different sampling times revealed no significant differences in richness (Chao 1 index), or other alpha-diversity indices (Shannon and Observed OTUs) between animals on ABs and NoABs treatment at the different sampling times (*p*-value > 0.05, Supplementary Figure 1). However, the alpha-diversity indexes reveal a notable difference between the cecal microbiota diversity depending on the time of sampling (weaning day *vs*. end of the growing period) (*p*-value < 0.05, Supplementary Figure 1).

Beta-diversity was measured by PERMANOVA test using Bray-Curtis dissimilarity and revealed a similar pattern to that of alpha-diversity, with no significant differences between the animals from ABs and NoABs groups (*p*-value > 0.05, Supplementary Figure 2). Results of the PERMANOVA indicated that there were significant differences in the microbial community composition in relation to sampling time (weaning day *vs*. end of the growing period) (*p*-value < 0.05, Supplementary Figure 2).

#### Taxonomic characterization of cecum microbial communities

To better understand how the microbial community composition changes between the different sampling times (weaning day *vs*. end of the growing period) and the management conditions (ABs *vs*. NoABs), we examined which organisms were present at different taxonomic levels and their relative abundance. Alignment of ASVs against the SILVA database resulted in identification of 9 bacterial phyla and 134 bacterial genera. While the majority of OTUs were identified at the genus level, some were only classified at the phylum, class, order or family.

**Figure 1:**
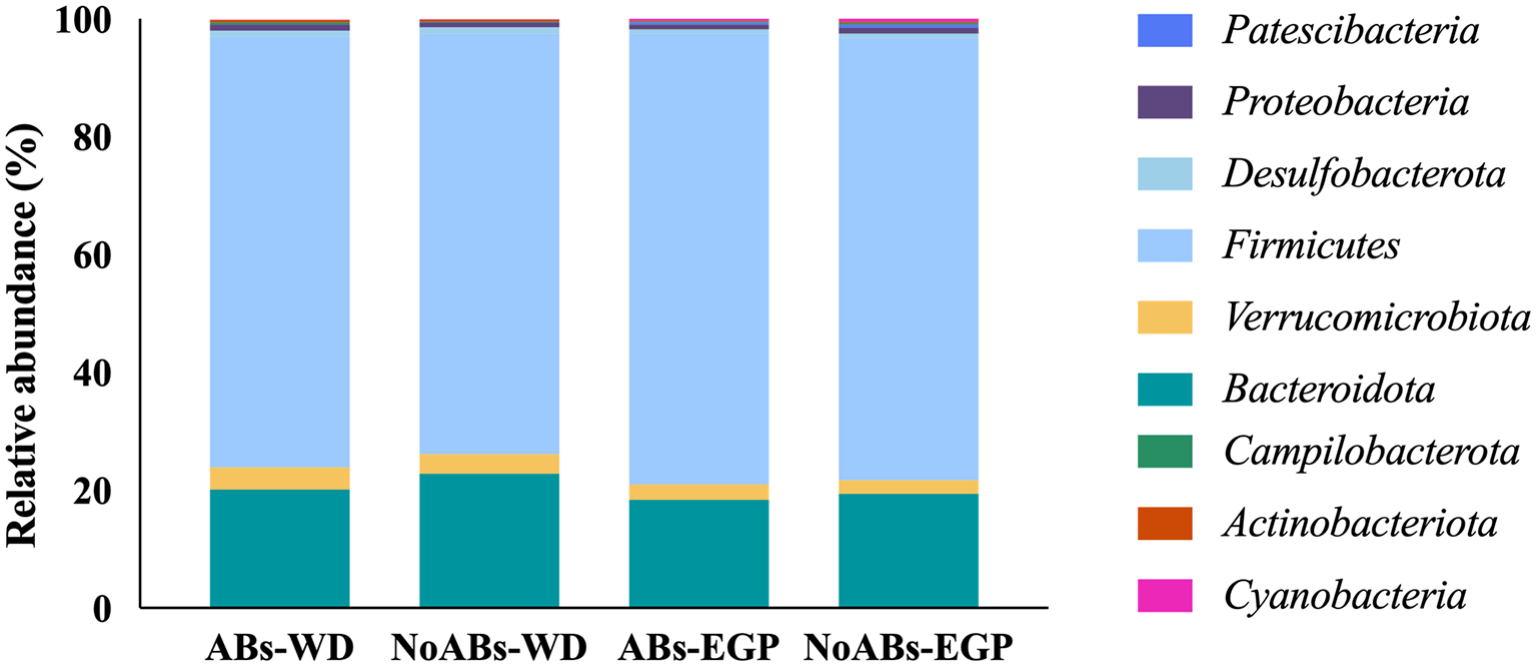
Taxonomic analysis at phylum level according to management conditions (ABs *vs*. NoABs) and sampling time (weaning day *vs*. end of the growing period). ABs-WD, animals fed with AB supplementation at weaning day; NoABs-WD, animals fed without AB supplementation at weaning day; ABs-EGP, animals fed with AB supplementation at the end of the growing period; NoABs-EGP, animals fed without AB supplementation at the end of the growing period.

At phylum level, *Firmicutes* represented the dominant phylum of the cecal community, followed by *Bacteroidota* in both groups at the two sampling times (Table 2; Figure 1).

**Table 2:** Relative abundance (%) of the taxonomic profiles in caecal samples at phylum level according to management conditions (ABs *vs*. NoABs) and sampling time (weaning day *vs*. end of the growing period).

**Phylum**	**Weaning day**		**End of growing period**	
	**ABs**	**NoABs**	**ABs**	**NoABs**
*Bacteroidota*	20.0	22.7	18.3	19.3
*Verrucomicrobiota*	3.9	3.4	2.7	2.4
*Firmicutes*	73.1	71.4	76.5	75.0
*Desulfobacterota*	1.0	1.1	0.7	0.8
*Proteobacteria*	1.0	0.8	0.8	1.0
*Patescibacteria*	0.0	0	0.4	0.5
*Campylobacterota*	0.4	0.1	0.1	0.3
*Actinobacteriota*	0.4	0.4	0.2	0.2
*Cyanobacteria*	0.1	0.1	0.3	0.6

ABs, rabbits fed with antibiotic supplementation; NoABs, rabbits fed without antibiotic supplementation.

**Figure 2:**
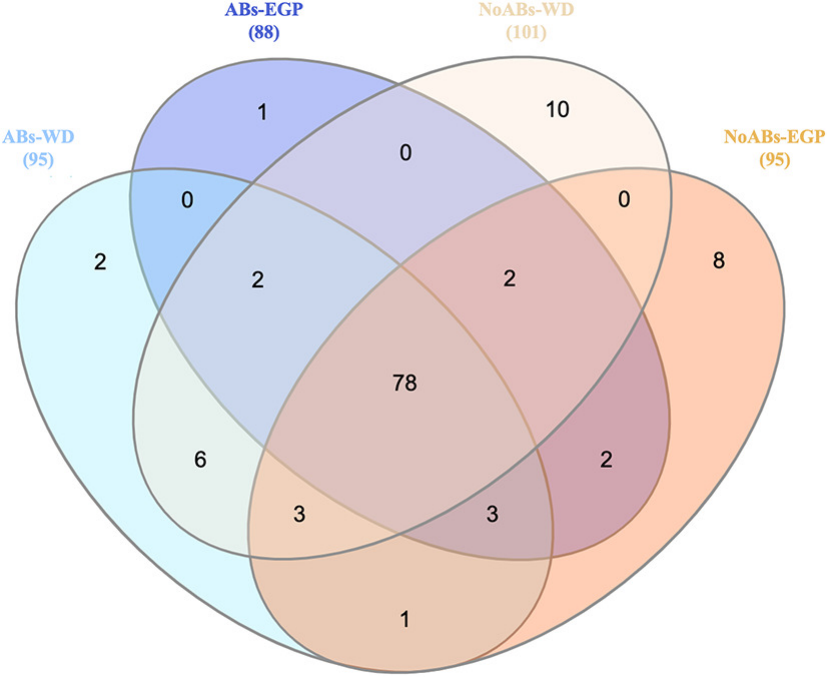
Venn diagram showing unique and shared taxa at genus level between samples according to antibiotic treatment (ABs *vs*. NoABs) and sampling time (weaning day *vs*. end of the growing period). Selection criteria were based on presence or absence regardless of abundance. Not drawn to scale. Value in brackets represent the genera found in each origin. Abs, rabbits fed with antibiotic supplementation; NoABs, rabbits fed without antibiotic supplementation; WD, weaning day; EGP, end of the growing period.

Among the 134 genera detected, 95 and 88 genera were detected in ABs group for the samples of the weaning day and the end of the growing period, respectively (Figure 2). Meanwhile, 101 and 97 genera were detected in NoABs group for the samples of the weaning day and the end of the growing period, respectively (Figure 2). Moreover, 78 genera were shared by groups (Figure 2). Finally, identified genera present only in NoABs group at the end of the growing period were *Victivallis* (0.01% of relative abundance), and *Escherichia-Shigella* (0.002% of relative abundance).

**Figure 3:**
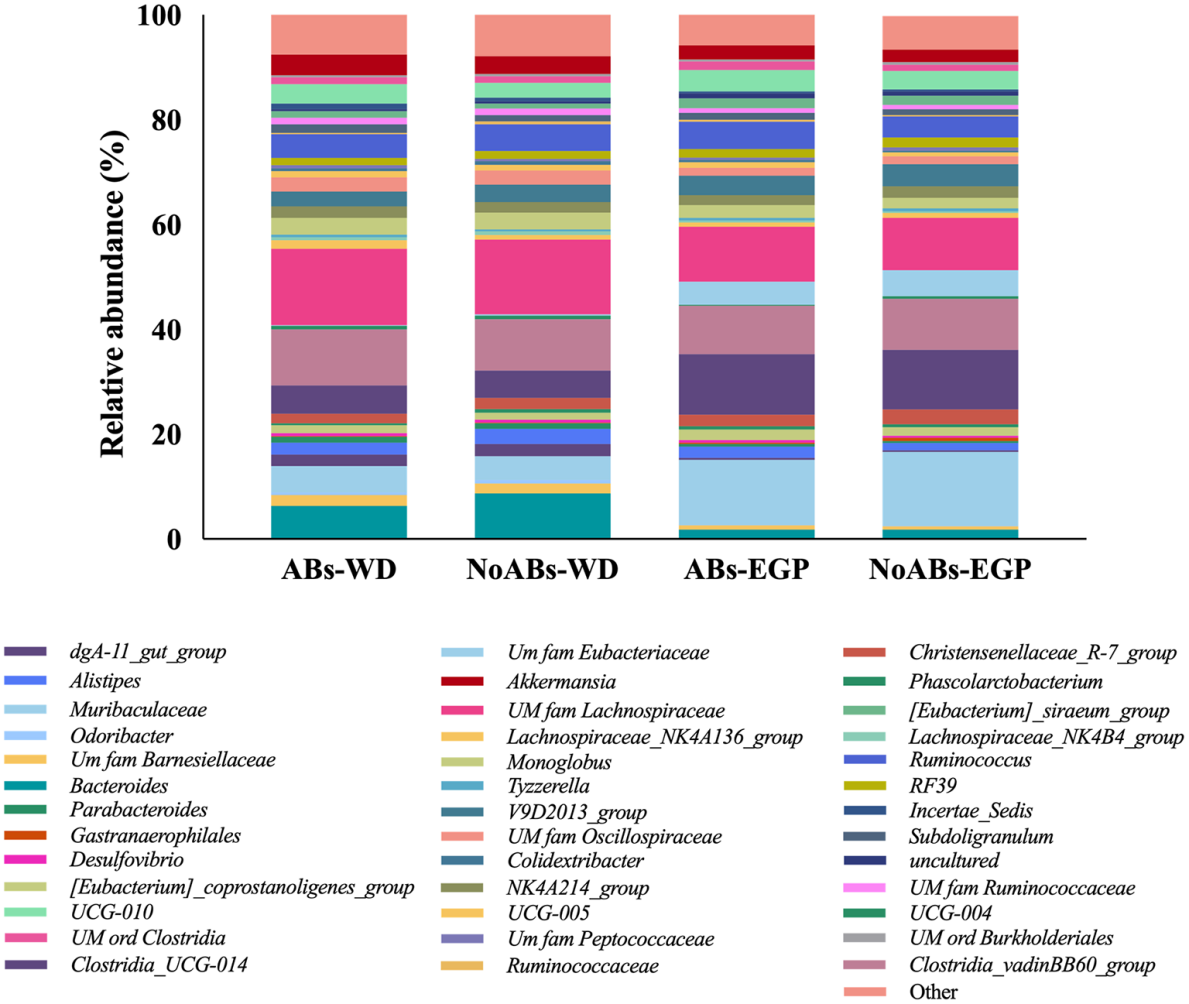
Taxonomic analysis at genus level according to management conditions (ABs *vs*. NoABs) and sampling time (weaning day *vs*. end of the growing period). ABs-WD, animals fed with AB supplementation at weaning day; NoABs-WD, animals fed without AB supplementation at weaning day; ABs-EGP, animals fed with AB supplementation at the end of the growing period; NoABs-EGP, animals fed without AB supplementation at the end of the growing period.

The 45 genera with a relative abundance of more than 0.5% in at least one sample group are presented in Supplementary Table 1 and Figure 3 (28, 33). In weaning day rabbits, for both experimental groups the most prevalent genera were unclassified members (U.m.) of *Lachnospiraceae* family (14.6 and 14.2%, respectively), *Clostridia_vadinBB60_group* spp. (10.7 and 9.8%, respectively), *Bacteroides* spp. (6.3 and 8.7%, respectively), *Clostridia_UCG-014* spp. (6.3 and 8.7%, respectively), *Muribaculaceae* spp. (5.0 and 4.5%, respectively) and *Ruminococcus* spp. (4.5 and 5.1%, respectively). Finally, at the end of the growing period, for both experimental groups the most common genera were *Muribaculaceae* spp. (12.4 and 14.1%, respectively), *Clostridia_UCG-014* spp. (11.6 and 11.4%, respectively), U.m. of *Lachnospiraceae* family (10.5 and 10.0%, respectively), *Clostridia_vadinBB60_group* spp. (19.2 and 9.7%, respectively), *Ruminococcus* spp. (5.2 and 4.0%, respectively) and U.m. of *Eubacteriaceae* family (4.4 and 5.0%, respectively).

ANCOM was performed between samples according to antibiotic treatment (ABs *vs*. NoABs) and sampling time (weaning day *vs*. end of the growing period). Weaning day had three differentially abundant taxa against end of the growing period: *Clostridia_UCG-014* spp. (W = 4,292), *Muribaculaceae* spp. (W = 4,143), and U.m. of *Eubacteriaceae* family (W = 4,048). In ABs and NoABs in sampling time did not yield differentially abundant taxa.

### Productive performance and health status

The productive parameters obtained were in accordance with the breed standards, without significant differences for any of the productive parameters measured between management systems (ABs *vs*. NoABs) (*p*-value > 0.05).

Regarding health status of the rabbits and diarrhea presence, significant differences were observed during the second week of the growing period, when NoABs group showed 6.20% of animals with diarrhea, while the ABs group only presented 1.05% (Table 3).

**Table 3:** Presence of diarrhea symptoms and mortality rates during the growing period for both experimental groups.

**Time**	**Management system**	**Diarrhea symptoms (%)**	**Mortality (%)**
wk 1	ABs	1.39	0
	NoABs	2.80	0.34
wk 2	ABs	1.05^a^	0.34^a^
	NoABs	6.20^b^	4.53^b^
wk 3	ABs	0.70	1.05^a^
	NoABs	3.09	4.74^b^
wk 4	ABs	0.71	0.70
	NoABs	0	0.77
wk 5	ABs	0	1.42
	NoABs	1.16	0.39

wk, week; ABs, rabbits fed with antibiotic supplementation; NoABs, rabbits fed without antibiotic supplementation.

a, b , different superscripts within the same column in the same week means significant differences (p-value < 0.05).

Finally, mortality rates showed significant differences between groups (*p*-value < 0.05) at the second (ABs group: 0.34%, and NoABs group: 4.53%) and third (ABs group: 1.05%, and NoABs group: 4.74%) weeks of growth (*p*-value < 0.05) (Table 3).

## Discussion

The present study assessed the cecal microbiota development and performance parameters in two different rabbit management systems during the growing period: animals fed with AB supplementation, and animals fed without AB supplementation. As reported above, although the increase in multidrug-resistant zoonotic pathogens in the food chain is one of the main concerns of public health, AB are still added in rabbit diets to prevent epizootic enteropathy (6). However, society and European health authorities are forcing the agri-food sector to find cost-effective, animal and environment friendly alternatives to AB.

In this sense, knowing the development of rabbit microbiota composition from weaning to the end of the growing period and how management practices, such as AB supplementation, influence its modulation could help in decision-making at farm level, and to evaluate the efficacy of alternatives proposed, such as robust genetic lines. For that reason, it might be interesting to consider microbiota composition as a biomarker of rabbit health and productive performance (15).

It is well demonstrated that a greater complexity of the cecal microbiota is observed as animals grow (34). Our findings showed that there is an important change in microbiota diversity from the weaning day to the end of the growing period, regardless of the administration of antibiotics at sub-therapeutic doses during the growing period, in agreement with previous studies (16, 35). Previous authors reported that microbial diversity is positively related to gut health (15, 16). In this sense, the results of this study showed that the withdrawal of ABs from the diet has no negative implications for intestinal health.

Regarding microbiota composition, *Firmicutes* and *Bacteroidota* were the predominant phyla for all groups, according to previous studies (15). *Firmicutes* has a fundamental role in rabbits' digestion, as it was considered the most efficient cellulose degrader (15, 36). Thus, the fact that no differences were found in this phylum between groups may mean that the absence of ABs in these animals would not affect the intestinal function of rabbits. Within this phylum, *Ruminococcus* spp. is the most relevant genus, being dominant in healthy rabbits and decreasing in the presence of disease (15, 37). Members of the *Lachnospiraceae* family are also significantly abundant in the microbiota of rabbits, as concurred with the findings of previous authors (35, 38, 39). High prevalence of this group has been observed in healthy young rabbits, related to the stimulation of cecotrophic behavior, which was also associated with a reduction in mortality (40). Moreover, *Bacteroidetes* was one of the major commensal phyla in the gut microbiota of rabbits, without statistical differences between groups. This phylum is proven to stimulate the development of gut-associated immune tissue (15, 36, 41). Indeed, members of this phylum have been related to degradation of vegetal polysaccharides and amino acid fermentation (16). Specifically, the presence of *Muribaculaceae*, the most relative abundant genus, has an important impact on host development and health (42). In this study, the removal of ABs in the feed had no impact on their abundance, maintaining a correct balance in cecal microbiota for both groups. On the other hand, the family *Clostridiaceae* plays an important role, being responsible for cellulose degradation, although the presence of some species in the cecum could lead in a decrease in butyrate yield, which has been related to rabbit epizootic enteropathy (16, 43). Although antibiotics have been used to control these bacteria (35), no differences were observed between groups.

However, at the end of the growing period it is important to highlight that *Victivallis* and *Escherichia-Shigella* genera, although in low relative abundances, were only present in the NoABs group, and both are associated with rabbit epizootic enteropathy (44, 45). For that reason, they could be considered as biomarkers of this disease, and alterative tools to AB should be developed and evaluated against these genera.

Regarding productive parameters, large group sizes during the growing period have been associated with negative effects on the mean daily gain, the mean daily intake, and the final body weight (46). Conversely, AB used as growth promoters have been related to better productive profitability (47). However, in this study, probably due to the use of a robust genetic line (LP), with greater resilience and better management of available resources (23), productive performance was according to the breed standards in both experimental groups. Thus, its development should be considered as an interesting alternative tool to reduce AB administration in rabbit production.

Finally, regarding health status of the rabbits and diarrhea presence, significant differences were observed during the second week of the growing period, when the NoABs group showed a higher percentage of diarrhea and mortality rate, probably associated with the effects of rabbit epizootic enteropathy (48, 49). This fact could be related to pathological disorders without a microbiota impact (50). However, it would be necessary to obtain global approaches to microbiability through the omic sciences (metagenomics, metabolomics, transcriptomics, genomics and epigenomics, etc.) throughout the growing period.

In conclusion, AB feed supplementation had no effect on microbiota diversity and phyla composition when a robust genetic line LP was reared. However, some differences appeared at genera level when antibiotics has been removed, probably related to rabbit epizootic enteropathy. For that reason, further studies are needed to pinpoint the specific causes of this disease and be able to develop complementary, effective, sustainable and animal-friendly alternatives applicable during the rabbit growing period to avoid antibiotic use on rabbit farms.

## Data availability statement

The datasets presented in this study can be found in online repositories. The names of the repository/repositories and accession number(s) can be found below: https://www.ncbi.nlm.nih.gov/, SAMN30645692.

## Ethics statement

The animal study was reviewed and approved by Ethical Review Panel of the Directorate-General for Agriculture, Fisheries and Livestock of the Valencian Community under code 2018/VSC/PEA/0067.

## Author contributions

Data curation: LM-D, LL-R, AR-M, MP-G, CM, and AV. Formal analysis: LM-D, LL-R, and MT. Funding acquisition: AV. Investigation: LM-D and CM. Methodology: AV and CM. Writing–original draft: LM-D, LL-R, and CM. Writing–review and editing: MP-G, CM, and AV. All authors contributed to the article and approved the submitted version.

## Funding

This research was funded by the National Institute for Agricultural Research and Experimentation and the Ministry of Economy, Industry and Competitiveness (RTA 2017-00013, Programme: MINECO, Ministerio de Economía y Competitividad). LL-R was supported by a research grant from the Generalitat Valenciana-Fondo SocialEuropeo (ACIF/2020/376).

## Conflict of interest

The authors declare that the research was conducted in the absence of any commercial or financial relationships that could be construed as a potential conflict of interest.

## Publisher's note

All claims expressed in this article are solely those of the authors and do not necessarily represent those of their affiliated organizations, or those of the publisher, the editors and the reviewers. Any product that may be evaluated in this article, or claim that may be made by its manufacturer, is not guaranteed or endorsed by the publisher.
