# Supplementary material for: Antibiotic removal does not affect cecal microbiota balance and productive parameters in LP robust rabbit line
Source: Front Vet Sci. 2022 Nov 7;9:1038218. doi: 10.3389/fvets.2022.1038218 (PMC9676498; doi:10.3389/fvets.2022.1038218)
Supplement: Supplementary file 1 [file Data_Sheet_1.docx]

Supplementary Material


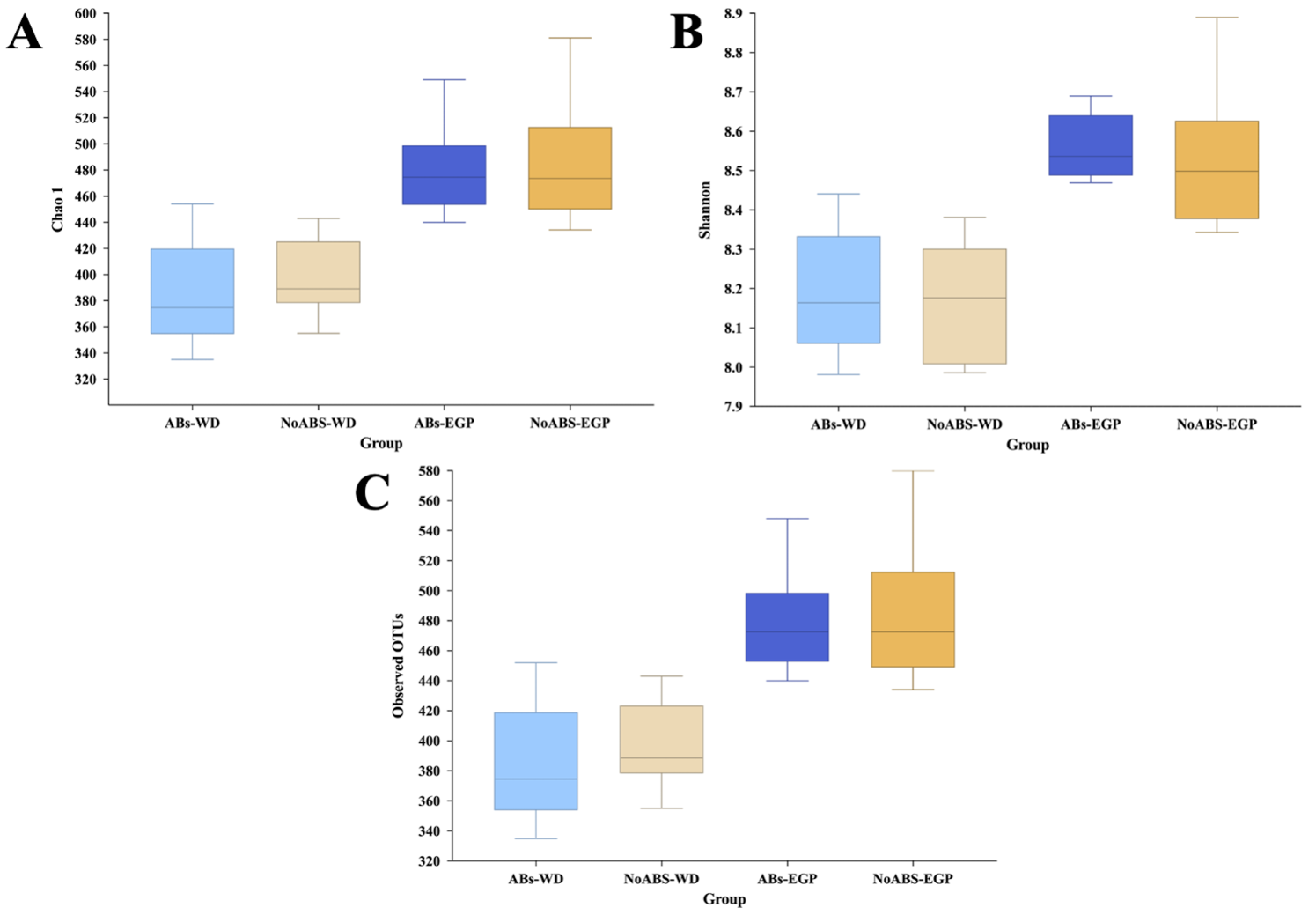


**Supplementary Figure 1.** Evaluation of alpha diversity in ABs and NoABs experimental groups by using different calculation measures: Chao 1, Shannon, and Observed Operational Taxonomic Units (OTUs). ABs-WD: rabbits fed with antibiotic supplementation at weaning day, NoABs-WD: rabbits fed without antibiotic supplementation at weaning day, ABs-EGP: rabbits fed with antibiotic supplementation at the end of the growing period, NoABs-WD: rabbits fed without antibiotic supplementation at the end of the growing period.


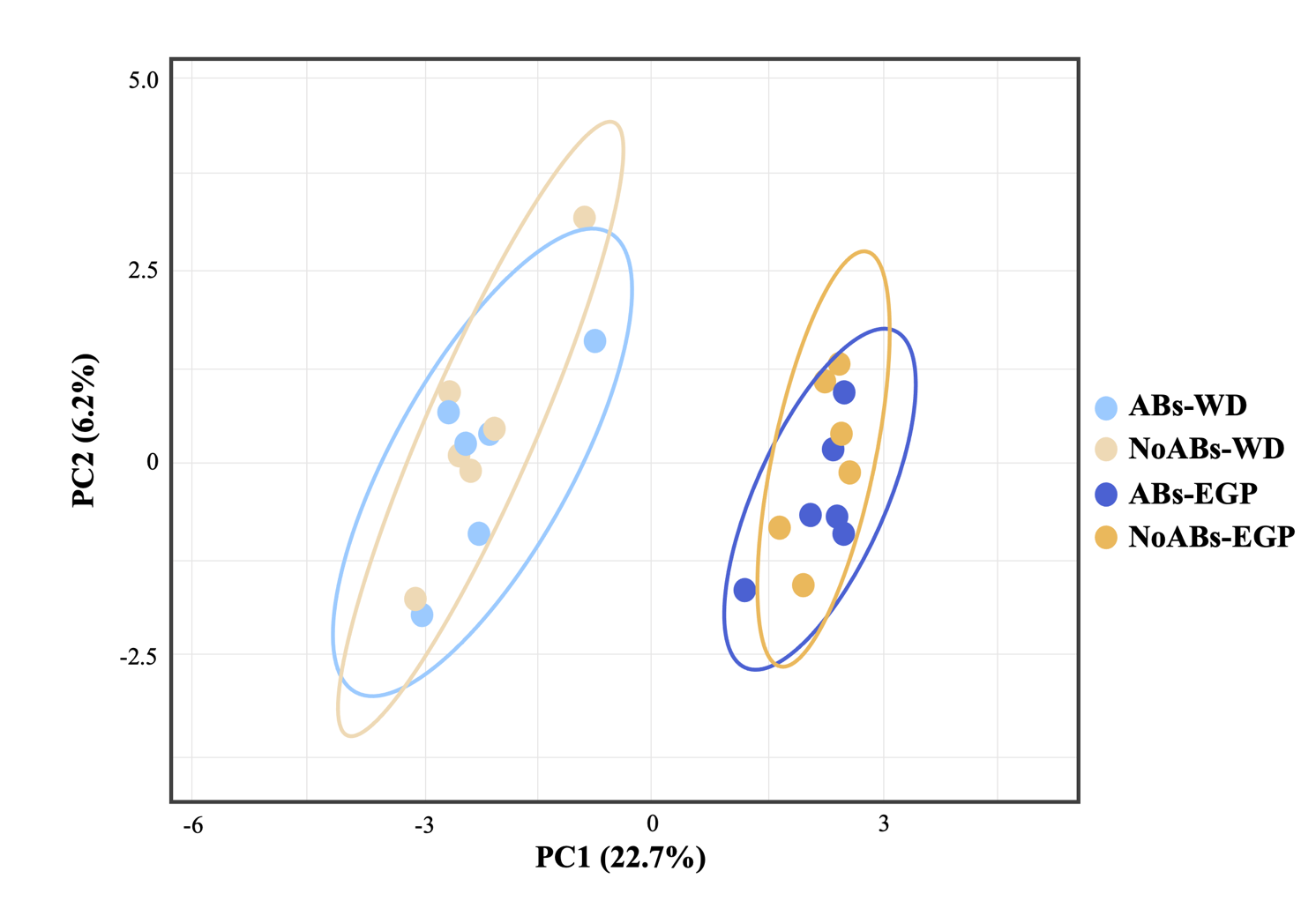


**Supplementary Figure 2.** Evaluation of the beta-diversity represented by PCoA graphic for ABs and NoABs experimental groups. ABs-WD: rabbits fed with antibiotic supplementation at weaning day, NoABs-WD: rabbits fed without antibiotic supplementation at weaning day, ABs-EGP: rabbits fed with antibiotic supplementation at the end of the growing period, NoABs-WD: rabbits fed without antibiotic supplementation at the end of the growing period.
