# Supplementary material for: Antibiotic removal does not affect cecal microbiota balance and productive parameters in LP robust rabbit line
Source: Front Vet Sci. 2022 Nov 7;9:1038218. doi: 10.3389/fvets.2022.1038218 (PMC9676498; doi:10.3389/fvets.2022.1038218)
Supplement: Supplementary file 2 [file Table_1.docx]

**Supplementary Table 1.** Relative abundance (%) of the taxonomic profiles at genus level according to management conditions (ABs *vs*. NoABs) and sampling time (weaning day *vs*. end of the growing period).

| **Phylum** | **Family** | **Genera** | **ABs-WD** | **ABS-EGP** | **NoABs-WD** | **NoABs-EGP** |
| --- | --- | --- | --- | --- | --- | --- |
| *Bacteroidota* | *Bacteroidaceae* | *Bacteroides* | 6.3 | 1.8 | 8.7 | 1.8 |
|  | *Barnesiellaceae* | *Um fam Barnesiellaceae* | 2.1 | 0.8 | 1.9 | 0.6 |
|  | *Marinifilaceae* | *Odoribacter* | 0.5 | 0.1 | 0.7 | 0.1 |
|  | *Muribaculaceae* | *Muribaculaceae* | 5.0 | 12.4 | 4.5 | 14.1 |
|  | *Rikenellaceae* | *dgA-11_gut_group* | 2.2 | 0.4 | 2.3 | 0.3 |
|  |  | *Alistipes* | 2.3 | 2.1 | 2.9 | 1.4 |
|  | *Tannerellaceae* | *Parabacteroides* | 1.1 | 0.5 | 1.1 | 0.4 |
| *Cyanobacteria* | *Gastranaerophilales* | *Gastranaerophilales* | 0.1 | 0.3 | 0.1 | 0.6 |
| *Desulfobacterota* | *Desulfovibrionaceae* | *Desulfovibrio* | 0.6 | 0.5 | 0.6 | 0.4 |
| *Firmicutes* | *[Eubacterium]_coprostanoligenes_group* | *[Eubacterium]_coprostanoligenes_group* | 1.5 | 2.0 | 1.3 | 1.6 |
|  | *Acidaminococcaceae* | *Phascolarctobacterium* | 0.4 | 0.6 | 0.7 | 0.6 |
|  | *Christensenellaceae* | *Christensenellaceae_R-7_group* | 1.8 | 2.2 | 2.1 | 2.8 |
|  | *Clostridia_UCG-014* | *Clostridia_UCG-014* | 5.4 | 11.6 | 5.2 | 11.4 |
|  | *Clostridia_vadinBB60_group* | *Clostridia_vadinBB60_group* | 10.7 | 9.2 | 9.8 | 9.7 |
|  | *Erysipelatoclostridiaceae* | *UCG-004* | 0.7 | 0.2 | 0.7 | 0.5 |
|  | *Eubacteriaceae* | *Um fam Eubacteriaceae* | 0.1 | 4.4 | 0.3 | 5.0 |
|  | *Lachnospiraceae* | *UM fam Lachnospiraceae* | 14.6 | 10.5 | 14.2 | 10.0 |
|  |  | *Lachnospiraceae_NK4A136_group* | 1.6 | 0.8 | 0.9 | 0.9 |
|  |  | *Lachnospiraceae_NK4B4_group* | 0.6 | 0.4 | 0.7 | 0.3 |
|  |  | *Tyzzerella* | 0.5 | 0.5 | 0.4 | 0.6 |
|  | *Monoglobaceae* | *Monoglobus* | 3.2 | 2.4 | 3.2 | 2.0 |
|  | *Oscillospiraceae* | *NK4A214_group* | 2.2 | 1.9 | 2.0 | 2.2 |
|  |  | *V9D2013_group* | 2.8 | 3.7 | 3.3 | 4.2 |
|  |  | *UM fam Oscillospiraceae* | 2.7 | 1.5 | 2.7 | 1.5 |
|  |  | *UCG-005* | 1.2 | 1.1 | 1.1 | 0.8 |
|  |  | *Colidextribacter* | 0.5 | 0.4 | 0.7 | 0.3 |
|  | *Peptococcaceae* | *Um fam Peptococcaceae* | 0.6 | 0.5 | 0.4 | 0.6 |
|  | *RF39* | *RF39* | 1.4 | 1.6 | 1.5 | 1.9 |
|  | *Ruminococcaceae* | *Ruminococcus* | 4.5 | 5.2 | 5.1 | 4.0 |
|  |  | *Ruminococcaceae* | 0.3 | 0.4 | 0.6 | 0.3 |
|  |  | *Subdoligranulum* | 1.6 | 1.3 | 1.2 | 1.1 |
|  |  | *UM fam Ruminococcaceae* | 1.3 | 0.9 | 1.2 | 0.8 |
|  |  | *[Eubacterium]_siraeum_group* | 1.2 | 1.9 | 1.0 | 1.8 |
|  |  | *uncultured* | 0.4 | 0.9 | 0.4 | 0.8 |
|  |  | *Incertae_Sedis* | 1.1 | 0.4 | 0.7 | 0.4 |
|  | *UCG-010* | *UCG-010* | 3.7 | 4.1 | 2.8 | 3.5 |
|  | *UM ord Clostridia* | *UM ord Clostridia* | 1.3 | 1.6 | 1.3 | 1.2 |
| *Proteobacteria* | *UM ord Burkholderiales* | *UM ord Burkholderiales* | 0.4 | 0.4 | 0.4 | 0.5 |
| *Verrucomicrobiota* | *Akkermansiaceae* | *Akkermansia* | 3.9 | 2.7 | 3.4 | 2.4 |

ABs-WD: rabbits fed with antibiotic supplementation at weaning day, NoABs-WD: rabbits fed without antibiotic supplementation at weaning day, ABs-EGP: rabbits fed with antibiotic supplementation at the end of the growing period, NoABs-WD: rabbits fed without antibiotic supplementation at the end of the growing period.
